# LEVERAGING PATIENT PORTALS TO IDENTIFY AND INTERVENE IN DEMENTIA CARE: CAREGIVING, MEDICATION, AND ACUTE CARE

**DOI:** 10.1093/geroni/igae098.0658

**Published:** 2024-12-31

**Authors:** Jennifer Portz, George Demiris

**Affiliations:** Chair; Discussant

## Abstract

Care partners play a pivotal role in supporting people living with dementia (PLWD), yet their systematic identification and involvement in clinical care are often lacking. This symposium presents four studies highlighting ground-breaking care partner identification methods to inform patient portal-based interventions targeting care partners to enhance dementia care. The first study, a retrospective cohort analysis focuses on automating care partner identification via structured patient contact data and unstructured portal notes in the electronic health record to better deliver evidence-based caregiving interventions through patient portals. The second study explores the associations between the documentation of a care partner and documentation of care partner education in the electronic health record and hospital length of stay. The third study presents an analysis of patient secure portal messages and clinical notes that identified modifiable contributors to potentially preventable acute care visits among PLWD including unmet care needs, particularly in care coordination, health systems navigation, and medication management that contribute. The final study describes the content of patient portal messages relating to medications among PLWD, care partners, and clinicians in a single health system, to inform the development of a portal-based intervention to reduce the use of potentially inappropriate medications among PLWD Our Discussant, Dr. George Demiris, PhD, an informatics and caregiving technology expert, will lead a conversation emphasizing the importance of systematic care partner identification and integrating care partner engagement tools with patient portals to enhance support for PLWD and their care partners, ultimately improving dementia care delivery and outcomes.

